# Emigration from the perspective of Polish health professionals – insights from a qualitative study

**DOI:** 10.3389/fpubh.2022.1075728

**Published:** 2022-12-23

**Authors:** Alicja Domagała, Aleksandra Kulbat, Kamila Parzonka

**Affiliations:** ^1^Institute of Public Health, Faculty of Health Sciences, Jagiellonian University Medical College, Krakow, Poland; ^2^Faculty of Medicine, Jagiellonian University Medical College, Krakow, Poland; ^3^Doctoral School of Medical and Health Sciences, Jagiellonian University Medical College, Krakow, Poland

**Keywords:** emigration, emigration drivers, health workforce, health professionals, human resources for health

## Abstract

The Polish health workforce is facing many problems, including shortages, heavy workload, burnout, and dissatisfaction, which may lead to job emigration, mainly among younger generations. The objective of the article is to explore the reasons, consequences, and circumstances of the emigration of Polish health professionals with a qualitative approach. We have conducted semi-structured interviews with 15 practicing health professionals, exploring the perspectives of different professions and different generations. The interviews were conducted using the on-line MS Teams platform from February until March 2022. We transcribed all interviews verbatim and performed directed qualitative content analysis. Currently, the scale of emigration is rather stable and mainly affects the younger generations. The main reasons for emigration, as indicated by representatives of different health professions, are similar, including excessive workload and long working hours. However, doctors and dentists also emphasized problems with professional development and specialization training, while nurses, midwives, and physiotherapists underlined problems with low prestige and work autonomy. Taking into account the substantial shortage of health workforce and the high average age of practicing health professionals, emigration requires special attention from health decision makers. Implementation of an effective mechanism for monitoring the emigration of medical staff is recommended.

## Introduction

In a globalized and interconnected, but significantly unequal world, health workforce migration is an important phenomenon and challenge for policymakers. The international mobility of health professionals has been increasing in the last two decades and forecasts indicate the further acceleration of this process (1–3). Due to population aging and the growing needs for health services, the demand for health workforce (HWF) is also increasing. The number of physicians and nurses has increased over the last decade in the majority of OECD countries, mainly due to growing numbers of national graduates, but foreign-born and foreign-trained professionals have also substantially contributed to this growth (2). Several countries have used international recruitment of medical staff to fill places in health facilities; however, this can intensify shortages and maldistribution in source countries (3, 4). The patterns of HWF migration are growing more complex, with significant intraregional movement (2); therefore, appropriate data are crucial for incorporating migration into an effective workforce planning approach (5, 6). There are essential regulations, reports, and initiatives of international organizations which could be helpful for the development of dedicated strategies, as this issue should be prioritized on national, European, and global health policy agendas (7–9).

Concerns about staff deficits and migration of HWF are not new in European countries. Directive 2005/36/EC of the European Parliament (10) makes HWF qualifications highly transferable across EU borders and guarantees the freedom of movement of the citizens. Professional qualifications achieved at EU universities are automatically recognized in other member states. The free mobility of HWF disproportionally advantages wealthier EU members at the expense of less advantaged countries, and that migration may feed disparities human resources flow from poorer to wealthier EU members (11). This situation raises equity concerns, and has consequences for setting health policy at the European level.

Currently, Poland is one of the countries where HWF situation is particularly unfavorable. According to OECD data, in 2020, Poland had one of the lowest numbers of practicing doctors and nurses per 1,000 population in the EU (12). Another problem is the increasing average age of practicing professionals (53 for nurses and 54 for specialist physicians). The current age structure of doctors and nurses leads a “generation gap,” resulting in the inability to replace older age groups with younger professionals entering the healthcare system. Further challenges of HWF include the low prestige of medical professions, difficult working and employment conditions, and unsatisfactory remuneration (13). The salaries of HWF are still low compared with western European countries. In 2019, doctors' average remuneration was 1.5 of the average remuneration in Poland and was the lowest among the 29 OECD countries for which data are reported (14). A common trend in Poland is the employment of health professionals simultaneously in more than one workplace. The average number of workplaces for doctors is 1.84, while it is 1.69 for midwives and 1.59 for nurses (13).

Deficits of HWF have been identified among the main groups of health professionals, and they are particularly significant in health facilities located in small cities and counties. According to the data reported by the Ministry of Family and Social Policy in the report, The Occupation Barometer 2022 (15), among 30 shortage occupations in Poland, 13 were health and care professions, including nurses, midwives, physicians, physiotherapists, paramedics, and carers of the elderly. Overall, there is a deficit of nurses and midwives in 347 (out of 380 analyzed) Polish counties and physicians in 317 (15). These data are also confirmed by research conducted in Polish hospitals before the COVID-19 pandemic which concluded that about 72% of hospitals reported a shortage of nurses, and 68% reported physician deficits (16). The current state of HWF in Poland is alarming, and the consequences of staff shortages are visible in many areas of healthcare system operations. These problems have been driven by many factors, including poor HWF management, inadequate planning in the medical education system, and also insufficient financing of the healthcare system.

Emigration of Polish medical staff had also occurred before Poland's EU accession (influenced mainly by geopolitical contexts, differences in salary, and working conditions), but the labor market has changed significantly, due to EU membership, since May 2004 (13). In Poland, as in many other countries, there is still no system to collect data and monitor the migration flow of HWF. The existing information is based only on the number of certificates issued by the medical chambers which confirm qualifications and the legal right to practice in other European countries. These data do not provide a picture of how many health professionals are actually working abroad; this should be used rather than as an estimation of the intention of medical workers to take a job in another European country (17).

In the case of Poland, migration of HWF mostly means emigration. The share of foreign-born health professionals is very low. According to OECD data, in 2019 the share of foreign-trained physicians was only 2.1%, which is much lower than the OECD average (17.9%). In the case of nurses, this indicator is marginal, assessed at around 0.1% (OECD average 6.1%) (14).

Several studies regarding the “brain-drain” of HWF have been conducted in Poland (13, 18–22), but there has been no systematic assessment of this phenomenon. “Brain drain” means the emigration of dissatisfied health professionals who are searching for a better standard of living, higher remuneration, better quality of life, easier access to advanced technology, and more stable socioeconomic conditions in different countries. The loss of highly qualified professionals has many negative consequences for Poland as a source country. In our previous study (13), we explored the trends and directions of emigration based on the information from national registers maintained by medical chambers and data analysis from the Regulated Profession Database in The EU Single Market. According to our findings, between 7 and 9% of doctors and nurses have applied for certificates confirming their right to practice in other European countries (most often the United Kingdom, Germany, Sweden, Spain, and Ireland) (13).

The aim of this research was to explore the reasons and circumstances of the emigration of Polish health professionals with a qualitative approach. Our contribution to the existing literature is based on new data, generated through qualitative research. A qualitative approach can better recognize the factors affecting migration intentions among medical staff, thus suggesting appropriate actions to reduce the scale of this phenomenon.

## Materials and methods

The methodology of this study was approved by the Bioethical Committee of the Jagiellonian University (approval number: 1072.6120.289.2021). The study, guided by the principle in scientific research to do no harm, gave the participants control over disclosure (23–25). Informed consent for research participation was obtained from all participants.

### Study design and setting

We conducted 15 semi-structured interviews with practicing health professionals, performing directed qualitative content analysis based on Hsieh and Shannon methodology, using the interview scenario as a guide (26). To ensure comprehensive reporting throughout this research, we used a checklist based on Consolidated criteria for reporting qualitative research (COREQ) (27) (Appendix 1).

Purposeful and snowball sampling were used to select interviewees representing the Polish health professionals (28). Respondents were selected for interview based on their knowledge/experience of the topic and active membership in the medical chamber. Potential respondents were recruited by email or phone. In selecting the potential participants, we tried to achieve gender and age balance (Table 1) to ensure the best possible insight with regard to the research aim. All invited respondents agreed to participate in our study. The interviews were conducted using the MS Teams platform from February until March 2022 and were carried out in the Polish language.

**Table 1:** Sociodemographic characteristics of the sample group.

	**Sex**	**Age group**	**Years of experience**	**Professional group**
1	F	40–45	17	Physiotherapist
2	M	40–45	16	Physician
3	M	25–30	2	Physician
4	F	40–45	17	Nurse
5	M	25–30	1	Physician
6	M	46–50	25	Paramedic
7	F	25–30	2	Nurse
8	F	25–30	3	Physician
9	M	40–45	15	Physiotherapist
10	F	50–55	27	Nurse
11	F	50–55	28	Dentist
12	F	40–45	20	Midwife
13	F	40–45	19 (2 years abroad) 02/2008–11/2009	Nurse
14	M	50–55	25 (15 years abroad) 11/2005–01/2021	Physician
15	M	56–60	33 (13 years abroad) 02/2007–04/2019	Dentist

There was no previous relationship between the interviewers and the participants. All members of the research team had previous experience with qualitative studies. Two authors of the article conducted the interviews (AK and KP). All three authors were involved in analyzing and interpreting the data. At the beginning of each interview, the researcher explained their professional background and presented a brief overview of the study and the objectives of the research. All respondents were assured that their names would remain confidential when publishing the study results. All names of our respondents, their organizations, and the full content of their responses remain undisclosed to ensure anonymity.

### Study population

Fifteen respondents participated in this study, and their sociodemographic characteristics are presented in Table 1. Seven respondents were male and eight female. Our respondents represent all the main professional groups in the healthcare system: physicians, dentists, nurses, midwives, physiotherapists, and paramedics. All respondents were competent and well-informed about the phenomenon of HWF emigration and have good knowledge of the healthcare system and its circumstances. Moreover, three of them have work experience in the medical profession abroad. We invited respondents with long professional experience (20–33 years) as well as representatives of younger generations. The number of our respondents was sufficient to reach thematic saturation (29).

### Data collection and analysis

An interview guide was developed in collaboration with the research group and the pilot was tested prior to the interviews with one physician and one nurse to ensure that questions were clear and appropriately adjusted. The same open-ended questions (without prompts or guidance) were repeated for all respondents, but the order of the questions varied depending on their responses and the circumstances of the interview. The interview guide consisted of 10 questions (Appendix 2) grouped around the four subthemes: (1) factors and reasons associated with the intention to emigrate; (2) main barriers to emigration; (3) benefits of emigration; (4) consequences of emigration. The interviews took between 40 and 50 min.

The interviews were recorded and transcribed verbatim. The texts of the interviews were read carefully and checked by two researchers to assure the highest accuracy. All three researchers were involved in analyzing the data and stated their opinions.

## Results

Fifteen health professionals were interviewed (Table 1). Different age groups were represented (28–60 years), with experience in their professions ranging from 1 to 33 years. This structure of the sample group allowed us to explore the perspectives of different generations.

The findings were clustered into four thematic subgroups:

### The key factors and reasons associated with the intention to emigrate

Almost all respondents, discussing the factors that lead to emigration, bring up issues of remuneration, at the same time stressing that this is the main, but not the only, reason for leaving. They also pointed to a lower workload abroad, a more precise definition of roles and division of tasks, linking remuneration with professional development, the opportunity to improve qualifications, and a better life-work balance. Many respondents stated there is no adequate systemic support for professional development, and that this problem applies to all medical professions.

*When I was leaving (2007), the biggest motivation was the wage gap and the ability to work using modern medical equipment. In my case, it was also an opportunity to ensure a better future for my children - education, learning English. Such a smooth start for children, easier and better professional development, but also curiosity about the world [dentist, M; 57 y; 13 y abroad]*.

Although we did not directly ask our respondents whether they were considering practicing their profession abroad, most of the younger respondents mentioned during the interview that due to the difficult working conditions (mainly workload, long working hours and administrative burden) they had considered such a decision in the past. Some respondents pointed out that motivational incentives clearly correlate to age:

*Financial factors were not the only thing that prompted me to leave. Due to globalization, a lot of relationships are mixed and this is also often a decision related to the family situation. In my case it was also a matter of broadening my horizons because it meant learning a living language, learning about the work patterns and standards that apply in the EU when it comes to nursing work [nurse, F; 41 y, 2 y abroad]*.

### The main barriers to emigration

The respondents indicated various emigration barriers, most often emphasizing language barriers and attachment to relatives and family, as well as the place of work and residence, but also the fear of moving to a new, unfamiliar life and working environment. According to the interviewees, currently, each of these obstacles is much less important than a dozen or so years ago. Former immigrants also noted the language barrier and organizational issues as the main difficulties:

*Obviously, language is the biggest barrier, especially in the case of phone communication, without direct personal contact with the patient. In addition, organizational issues; you need to know about referrals to specialists, to hospitals, for medical examinations [physician, M, 55 y, 15 y abroad]*.

Several respondents pointed to the age of emigrants:

*The barriers mainly affect older professionals. When it comes to young people, there are practically no such obstacles. Currently, the level of language knowledge among graduates is very advanced. Also, in younger generations, the frequent lack of family commitments and the blurring of cultural boundaries make it easier to decide to leave [physiotherapist, F; 42 y]*.

A nurse who worked in Ireland for 2 years notes that companies recruiting health professionals try to help them overcome various barriers in order to facilitate the adaptation process, including registration in the chamber of nurses, finding accommodation, and new job positions in hospitals.

### The benefits of emigration

The respondents indicated quite a long list of benefits for emigrants, including higher remuneration, better possibility of professional development, the opportunity to choose a medical specialization, shorter working time (fewer hours spent at work gives time for learning and development), paid leave, e.g., scientific leave, i.e., time that can be devoted to one's own scientific development. Moreover, the majority of the respondents also listed access to modern technologies, professional development related to using innovative equipment, the civil security of medical staff, the possibility of experiencing a different type of work organization, and self-improvement. Many participants stated that usually, benefits are in line with the pull factors of emigration.

*Greater prestige. Access to modern technologies and equipment allows you to broaden your horizons and allows you to conduct research and experiments, which is much more difficult in Poland. Also, at the same time – the awareness that you are doing something important, that you are developing and helping people, and that someone appreciates you for it. [nurse, F; 26 y]*.

*Wages and working conditions are the main benefits. A medical worker should not exceed the safe standard of effort, both physical and mental. His mind should always be ready to react quickly in an emergency [paramedic, M; 47 y]*.

The assessment of the benefits of emigration expressed by those respondents who had actually worked abroad is interesting. They stated:

*Opening up to international nursing – that's necessary. I always recommend everyone to go abroad because it really opens up horizons and systematizes certain issues; it also shows where to look and what can be changed. It also introduces a lot to our nursing, many positive aspects when it comes to practicing this profession. [nurse, F; 41 y, 2 y abroad]*.

*One of the greatest benefits I have gained is that they taught me to think economically, which is generally financial thinking - how much it costs [dentist, M; 57 y,13 y abroad]*.

### The consequences of emigration for the healthcare system

Emigration brings benefits to people who have emigrated, but it also results in numerous negative consequences for the healthcare system in Poland. Many participants stated that the main consequences are fewer employees in the system, which translates into more work and responsibilities, increasing staff frustration, and excessive workload, often leading to exhaustion and burnout. Many respondents also emphasized low employment indicators, which translates into longer waiting times for healthcare services: “*queues, queues, and again queues to specialists. Some patients move to the private sector, but not everyone can afford a private visit”*.

The respondents pointed out that staff shortages are particularly noticeable in smaller, district hospitals, where the demand for medical services remains unsatisfied. This applies mainly to a deficit of narrow specializations (e.g., pediatric cardiology/pediatric oncology). They also emphasized that excessive work and duties resulted in a lack of time for proper patient care. They drew attention to the fact that since young health workers leave, this also affects the age structure and aging of the working population in the country.

*The consequences are also visible in patient care and patient education because mainly only the most necessary tasks are done, those that are life-saving activities. A nurse does not have time to use the skills and knowledge that she acquired during her training and work experience because she does not have enough time for it [nurse, F; 41 y]*.

*The Polish state pays for the education of doctors who later emigrate - this is a big loss for the system. There are no hands to work, and education has been pre-paid from the public budget. This leads to the need to establish priority specializations, with better remuneration, to encourage doctors to choose the most needed specializations. [physician, M; 28 y]*.

Finally, we asked about actions that could be undertaken to reduce the scale of medical staff emigration. The respondents pointed out two key issues: (1) appropriate remuneration in the basic workplace, so there would be no need to work in several facilities, and (2) better opportunities for professional development. Many respondents emphasized the need to reduce excessive work by introducing support by other staff e.g., medical secretaries or caregivers.

*The most important issue for me is working time. You can of course earn good money, but how many hours do you have to work to get it? This often comes at the disadvantage of relationships and time spent with family, we are never home - it is a matter of life choices [dentist, M; 57 y; 13 y abroad]*.

*It is also important to focus on prestige and encourage young people to study nursing. So you have to show the possibilities and that nursing does not necessarily come down to working directly with patients, at the hospital bed, but that it is a profession that allows you to have a PhD degree and deepen your knowledge and find the right place in the system. [nurse, F; 41 y, 2 y abroad]*.

*Burnout should be prevented by maintaining certain standards - limiting the number of patients per physiotherapist and working time. [physiotherapist, F; 42 y]*.

The perspective of the representatives of the younger generations is interesting, as they pointed out that many health workers lack mentoring and support in professional development. Moreover:

*Changing the narrative in society is crucial. There is a belief that any medical profession must come with some kind of sacrifice. There are many unpleasant and hurtful comments that discourage you from continuing to work. In the comments on forums or web portals, one can notice a lot of hatred of society toward doctors, which is in painful opposition to the years of youth devoted to studies and science [physician, M; 28 y]*.

## Discussion

The aim of this study was to investigate circumstances and factors associated with intentions to migrate among Polish health personnel. We have explored opinions about the reasons, barriers, and consequences of emigration from the point of view of practicing medical professionals. The scale and trend of emigration is rather stable. Currently, mainly younger generations are interested in emigration and are considering leaving the country. These findings are in line with data reported by the national chambers of health professionals, according to which 7–9% of practicing physicians and nurses have applied for certificates confirming their legal right to practice in other European countries (13), the majority of those certificates having been issued just after Poland's accession to the EU. The downward trend in the subsequent years reflects the substantial increase in salary in Poland following the strikes of physicians in 2006/2007 and 2017/2018, and nurses' protests in 2015/2016 (5, 13). In 2017, after the next waves of doctors' strikes, the government adopted a legal regulation that would gradually increase medical workers' minimum salary (30). Despite the fact that the average salaries of HWF have increased during the last decade, health professionals are still not satisfied with their wages which are still much lower than in western EU countries.

We have found interesting results on the barriers to emigration. In the opinion of our respondents, these barriers mainly affect professionals in older age groups. In the case of young people “*there are practically no such barriers*”. Currently, the level of foreign language competencies among graduates is really high and younger generations are more open to life changes and working in a new culture and new working environment.

Our study has shown that younger generations are much more interested in emigration compared to older groups of professionals. Also, other research has confirmed that the younger generation of HWF place a higher value on their work-life balance (18, 31, 32). They demand flexibility in working time and shorter working hours (33), and they are interested in professional development, but they expect support and dedicated training leave. This requires changes in HWF management, including implementation of better motivation incentives and effective retention of young professionals.

The main emigration drivers indicated by our respondents were unsatisfactory salary, heavy workload, long working hours, unclear task division among professions, problems with professional development, and lack of work-life balance. It should be emphasized that the opinions expressed by our respondents represent different health professions (doctors, dentists, nurses, midwives, physiotherapists, and paramedics) but they were quite similar. However, physicians and dentists focused mainly on problems with professional development and specialization training, while respondents representing other groups underlined lack of prestige and work autonomy.

Humpries et al. (34) also reported that the main factors driving the migration of Irish physicians, nurses, and midwives are better working conditions, clearer career path and a better practice environment (34). Similar factors have also been reported in other European studies (6, 9, 11).

There is a clear consensus in the scientific research and literature that HWF migration has negative effects on the source country and its citizens, while the destination country and the individual health professional both benefit (1). Health professionals who have emigrated can benefit from working in their new environment thanks to communication and cooperation with patients, healthcare institutions, and colleagues from different cultures. Facing different new situations in multicultural environments, they could develop their attitude toward new systems and intra-personal relations with colleagues (35). Our respondents listed benefits such as better salary abroad, better possibility of professional development, shorter working time, access to modern technologies, and better work-life balance.

Migration is an integral part of the European labor market, expanding employment opportunities for individuals, but in the healthcare sector, close attention must be paid to the potential, unintended consequences of unequal HWF mobility and “brain drain”. Unbalanced distribution of medical staff can intensify health inequalities and generate problems of limited access to healthcare services (36). The emigration of HWF will continue; emigration routes will run along regions and places where it is easier to work or where the benefits of employment prevail (1, 2). There is a need for actions and interventions at the European level to diminish the negative effects and maximize the positive effects of free migration, mainly for the most vulnerable health systems in the EU, e.g., by developing a mobility database, coordinating medical training capacity, and providing financial and technical support to disadvantaged source countries (11). The emigration needs to be addressed properly by health policy actions; otherwise, the health system will lose efficiency due to staff shortages. In the context of EU priorities, existing HWF emigration and “brain drain” require special attention. Responses to the challenges raised by the emigration of HWF are country-specific and there are no good practices that can simply be replicated in other countries.

Recognizing and understanding the factors of Polish health professionals' intention to emigrate is important for medical associations, healthcare managers, and policymakers. Increasing HWF deficits, heavy workload, and poor working conditions may increase the frustrations and burnout of professionals and, as a consequence, intensify their intentions to emigrate or their decision to leave the medical profession. These factors need to be addressed by health decision makers if Poland wants to retain and build the capacity of its HWF.

The number of foreign-born health professionals in Poland is still very low (2). In 2020, due to the COVID-19 pandemic, a new legal act was introduced to strengthen HWF capacity by simplifying the procedure for the employment of HWF from non-EU countries.

Russian military aggression against Ukraine in February 2022 resulted in massive immigration of Ukrainian citizens to Poland, including health professionals. At the time of writing, more than 6.28 mln refugees have come to Poland and about 1.4 mln have been recorded and registered in temporary protection schemes (37). This situation necessitates the adjustment of the provisions regulating the health labor market, adapting it to the current challenging situation.

Our study provides new insights into emigration trends and reasons from the point of view of medical staff. We analyzed this phenomenon from the perspectives of different health professions and different generations, and we have included in our study professionals who are experienced in working abroad, which is important and brings interesting added value. To explore more deeply the nature of HWF migration, further research is needed. Including the voices of those professionals who have emigrated permanently could bring valuable new perspectives.

Our study is not free of limitations. The comparatively small sample size is one. This makes it difficult to formulate definitive conclusions and is insufficient to be properly representative of the Polish system. Nevertheless, there is evidence in the literature that even six interviews are enough to define the broad themes of any phenomenon (38). Moreover, greater participation in the research by health professionals with work experience in the medical profession abroad could have expanded and deepened the content of our analysis.

Taking into account the significant shortages of HWF and high average age of practicing professionals in Poland, the emigration phenomenon requires scrupulous monitoring and the special attention of policymakers. The qualitative approach allowed us to analyse the problem of and reasons for emigration. To investigate the nature of the phenomenon more deeply, further nationwide survey research is recommended to assess the scale and trends of migration intentions among Polish medical staff including different professional groups.

## Data availability statement

The datasets presented in this article are not readily available because the full content of the responses of our respondents remain undisclosed to ensure anonymity. Requests to access the datasets should be directed to AD, alicja.domagala@uj.edu.

## Ethics statement

The studies involving human participants were reviewed and approved by the Bioethical Committee of the Jagiellonian University (approval number: 1072.6120.289.2021). Written informed consent for participation was not required for this study in accordance with the national legislation and the institutional requirements.

## Author contributions

AD, AK, and KP: methodology, investigation, data analysis, findings interpretation, manuscript writing, and revision. AD: conceptualization and supervision. All authors meet the authorship criteria, agree to the submission of the manuscript, have made substantial contributions to the conception or design of the work, according to the International Committee of Medical Journal Editors (ICMJE), and to the Committee on Publication Ethics (COPE).
