# Supplementary material for: Emigration from the perspective of Polish health professionals – insights from a qualitative study
Source: Front Public Health. 2022 Dec 23;10:1075728. doi: 10.3389/fpubh.2022.1075728 (PMC9822540; doi:10.3389/fpubh.2022.1075728)
Supplement: Supplementary file 1 [file Data_Sheet_1.PDF]

Consolidated criteria for reporting qualitative studies (COREQ): 32-item checklist

Developed from: Tong A, Sainsbury P, Craig J. Consolidated criteria for reporting qualitative research (COREQ): a 32-item checklist for interviews and focus groups. International Journal for Quality in Health Care. 2007. Volume 19, Number 6: pp. 349 – 357.

YOU MUST PROVIDE A RESPONSE FOR ALL ITEMS. ENTER N/A IF NOT APPLICABLE

No. Item	Guide questions/description	Response
**Domain 1: Research team and reflexivity**		
Personal Characteristics		
1. Inter viewer/facilitator	Which author/s conducted the interview?	Kamila Parzonka, Aleksandra Kulbat
2. Credentials	What were the researcher's credentials? E.g. PhD, MD	Kamila Parzonka, MSc, PhD Candidate<br>Aleksandra Kulbat, MD
3. Occupation	What was their occupation at the time of the study?	Kamila Parzonka, PhD Student at the Doctoral School of Medicine and Health Sciences<br>Aleksandra Kulbat, Medical Doctor
4. Gender	Was the researcher male or female?	Female (2)
5. Experience and training	What experience or training did the researcher have?	Extensive qualitative experience through education and additional training prior the study.
Relationship with participants		
6. Relationship established	Was a relationship established prior to study commencement?	No relationship with interviewers prior to the study.
7. Participant knowledge of the interviewer	What did the participants know about the researcher? e.g. personal goals, reasons for doing the research	Prior to each interview, respondents were informed about the overview of the study and the objectives of the research (exploration the nature, reasons, circumstances and benefits of the emigration).
8. Interviewer characteristics	What characteristics were reported about the interviewer/facilitator? e.g. Bias, assumptions, reasons and interests in the research topic	Interviewers are PhD candidate and a medical doctor involved in the study on behalf of the Institute of Public Health. Participants were informed that the study protocol was approved by the Bioethical Committee of the Jagiellonian University.
**Domain 2: study design**		
Theoretical framework		

9. Methodological orientation and Theory	What methodological orientation was stated to underpin the study? e.g. grounded theory, discourse analysis, ethnography, phenomenology, content analysis	In this qualitative study we used content analysis.
Participant selection		
10. Sampling	How were participants selected? e.g. purposive, convenience, consecutive, snowball	Purposive sampling was used. Participants were selected based on their knowledge and experience of the topic and active membership in health professionals' association. The participation in the study was voluntary.
11. Method of approach	How were participants approached? e.g. face-to-face, telephone, mail, email	Participants were approached through telephone or email.
12. Sample size	How many participants were in the study?	15 participants were interviewed.
13. Non-participation	How many people refused to participate or dropped out? Reasons?	No one refused to participate.
Setting		
14. Setting of data collection	Where was the data collected? e.g. home, clinic, workplace	The interviews were conducted and data was collected using the on-line MS Teams platform
15. Presence of non-participants	Was anyone else present besides the participants and researchers?	No. Only participant and interviewer were present.
16. Description of sample	What are the important characteristics of the sample? e.g. demographic data, date	Representatives of practicing Polish health professionals, including: doctor, dentist, nurse, midwife, physiotherapist and paramedic. Seven were male and eight female. Three participants have work experience abroad.
Data collection		
17. Interview guide	Were questions, prompts, guides provided by the authors? Was it pilot tested?	The interview guide consisted of 10 questions and the pilot was tested prior to the study with 2 participants.
18. Repeat interviews	Were repeat interviews carried out? If yes, how many?	None
19. Audio/visual recording	Did the research use audio or visual recording to collect the data?	The interviews were audio recorded and transcribed verbatim.
20. Field notes	Were field notes made during and/or after the interview?	No
21. Duration	What was the duration of the interviews or focus group?	The interviews took between 40-50 minutes (min. 32 and max. 60).
22. Data saturation	Was data saturation discussed?	Yes
23. Transcripts returned	Were transcripts returned to participants for comment and/or	No

	correction?	
**Domain 3: analysis and findings**		
Data analysis		
24. Number of data coders	How many data coders coded the data?	1
25. Description of the coding tree	Did authors provide a description of the coding tree?	No
26. Derivation of themes	Were themes identified in advance or derived from the data?	Themes were identified from the interview guide topics and then further finalized from collected data
27. Software	What software, if applicable, was used to manage the data?	N/A
28. Participant checking	Did participants provide feedback on the findings?	No
Reporting		
29. Quotations presented	Were participant quotations presented to illustrate the themes/findings? Was each quotation identified? e.g. participant number	Yes, see section “Results”. In each quotation we provided information about profession, sex and age.<br>Participants numbers in the article were removed.
30. Data and findings consistent	Was there consistency between the data presented and the findings?	Yes
31. Clarity of major themes	Were major themes clearly presented in the findings?	Yes
32. Clarity of minor themes	Is there a description of diverse cases or discussion of minor themes?	Yes

Once you have completed this checklist, please save a copy and upload it as part of your submission. When requested to do so as part of the upload process, please select the file type: Checklist. You will NOT be able to proceed with submission unless the checklist has been uploaded.
