# Supplementary material for: Emigration from the perspective of Polish health professionals – insights from a qualitative study
Source: Front Public Health. 2022 Dec 23;10:1075728. doi: 10.3389/fpubh.2022.1075728 (PMC9822540; doi:10.3389/fpubh.2022.1075728)
Supplement: Supplementary file 2 [file Data_Sheet_2.PDF]

Appendix 2. Interview Questions

1. What are the main sources of information regarding emigration among Polish medical personnel?
2. What do we know about the scale and trends of this phenomenon?
3. What are the main factors that lead Polish health professionals to decide to emigrate?
4. What are the most common barriers/obstacles of emigration among health professionals?
5. What are the benefits for those health professionals who have decided to emigrate?
6. What are the consequences of emigration for the healthcare system?
7. Can we effectively mitigate emigration? If so, in what way?
8. Can we encourage the return of health professionals who have decided to work abroad?
9. Are you aware of mechanisms/practices that allow for efficient monitoring of medical staff migration?
10. What actions should be taken to establish an effective system for monitoring the emigration process?
